# Insights into novel antimicrobial compounds and antibiotic resistance genes from soil metagenomes

**DOI:** 10.3389/fmicb.2014.00489

**Published:** 2014-09-18

**Authors:** Alinne P. de Castro, Gabriel da R. Fernandes, Octávio L. Franco

**Affiliations:** ^1^Programa de Pós-Graduação em Biotecnologia, Universidade Católica Dom BoscoLaboratórios Inova, Campo Grande, Brazil; ^2^Programa de Pós-Graduação em Ciências Genômicas e Biotecnologia, Centro de Analises Proteomicas e Bioquimicas, Universidade Católica de BrasíliaBrasilia, Brazil

**Keywords:** antibiotics, metagenome, soil, resistance genes, drug development

## Abstract

In recent years a major worldwide problem has arisen with regard to infectious diseases caused by resistant bacteria. Resistant pathogens are related to high mortality and also to enormous healthcare costs. In this field, cultured microorganisms have been commonly focused in attempts to isolate antibiotic resistance genes or to identify antimicrobial compounds. Although this strategy has been successful in many cases, most of the microbial diversity and related antimicrobial molecules have been completely lost. As an alternative, metagenomics has been used as a reliable approach to reveal the prospective reservoir of antimicrobial compounds and antibiotic resistance genes in the uncultured microbial community that inhabits a number of environments. In this context, this review will focus on resistance genes as well as on novel antibiotics revealed by a metagenomics approach from the soil environment. Biotechnology prospects are also discussed, opening new frontiers for antibiotic development.

## INTRODUCTION

Nowadays one of the most intractable worldwide health problems involves treating infections that are resistant to antibiotics. Resistant pathogens are able to cause high mortality and consequently impose huge healthcare costs (Carlet et al., 2011). Until recently, antibiotics were used only for treating human infections. Now, however, antibiotics are being extensively used in agriculture, food industries, or veterinary practices, causing a high impact on natural environments and consequently on human health (Radhouani et al., 2014). This situation of *bacterial* gene resistance created by widespread and imprudent use of antibiotics has triggered a more energetic search for alternative compounds with deleterious activities against microbial infectious diseases, as well as the identification of pathways or genes related to resistance to traditional antibiotics.

In this regard, over the last decade, the developments of culture-independent approaches have allowed additional insights into the diversity of antimicrobial compounds and antibiotic resistance genes from different environments. Basically, culture-independent analyses are based on molecular methods, including the extraction, amplification, sequencing, and analysis of nucleic acids from environmental samples. Among these, the metagenomics approach has revolutionized knowledge about the vast majority of not-yet-culturable microbial communities. This idea, coined by Handelsman et al. (1998), briefly consists of direct or indirect DNA extraction from a microbial community in its natural habitat, bypassing microbial isolation, and traditional culturing methods.

**FIGURE 1:**
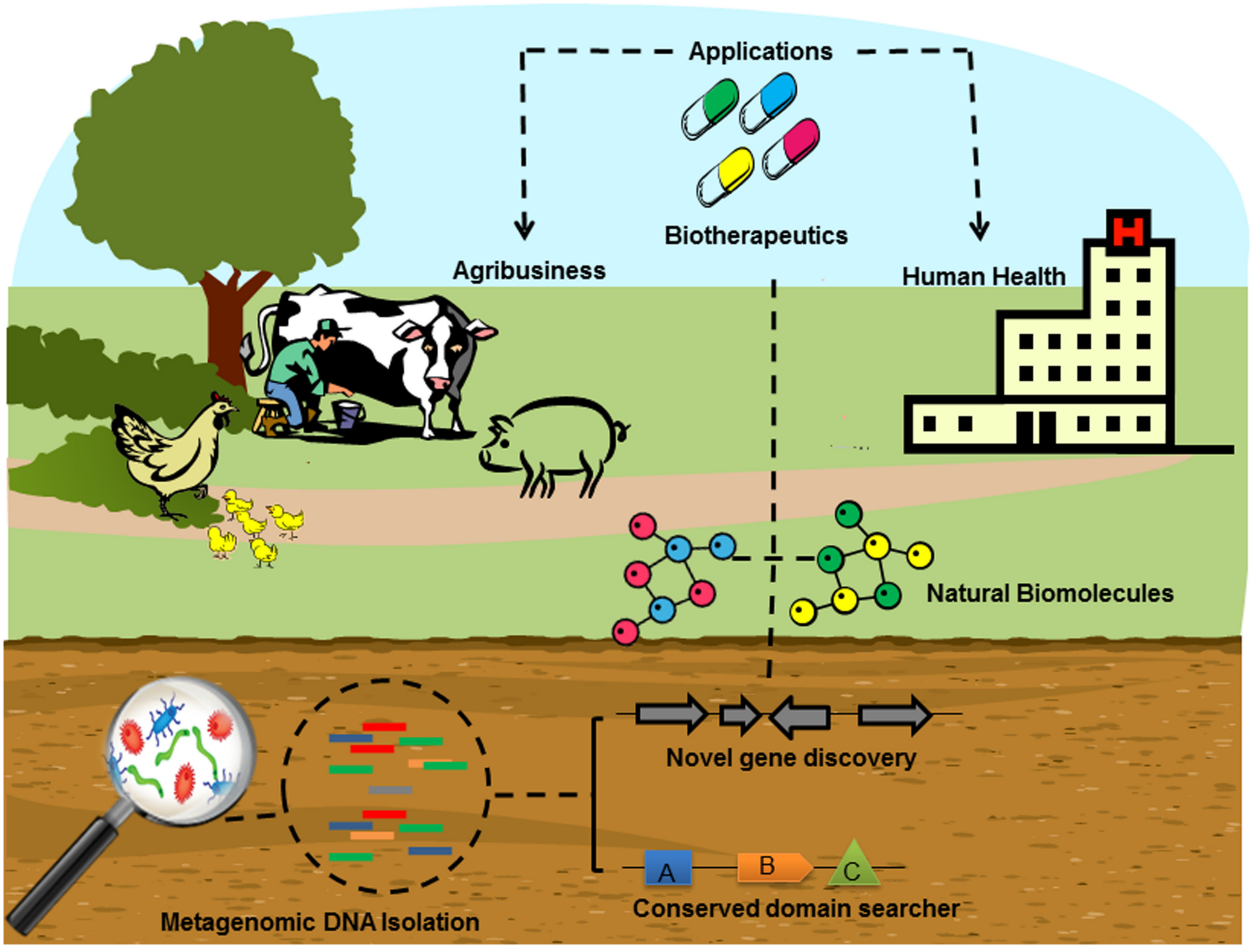
**Schematic depiction of the typical screening for novel antimicrobial compounds and antibiotic resistance genes from the soil environment through metagenomics.** After collecting soil samples, the metagenomic DNA is extracted and sequenced from a microbial community in its natural habitat, bypassing microbial isolation and traditional cultivation methods, generating several million reads. Once coding sequences have been obtained, their corresponding antimicrobial compounds can be sought through conserved domain search or novel gene discovery in the reference functional databases by *in silico* analysis. Complementary methods reconstruct the identification of the biomolecules of interest. Large-scale production of the target molecule is then carried out for various biotechnological applications including agribusiness and human health.

In recent decades, cultured microorganisms were the exclusive source from which to isolate and clone antibiotic resistance genes or identify antimicrobial activity, since most of the microbial diversity was lost when researchers tried to grow them in standard laboratory culture medium (Hugenholtz et al., 1998). For this reason, metagenomics is a reliable alternative approach to reveal the potential reservoir of antimicrobial compounds and antibiotic resistance genes in the uncultured microbial community that inhabits the environments (**Figure 1**).

Metagenomics has also been considered a promising approach for the isolation of unusual antibiotics from environmental samples, as well as in identifying the mechanisms of *bacterial* resistance in the *in situ* microbial community. The combination of metagenomics with next-generation deep sequencing has brought great progress to the field of antimicrobial resistance and compounds, giving a more feasible representation of the origins and mechanisms of resistance genes (Forsberg et al., 2012; McGarvey et al., 2012). Although the metagenomic approach has many applications in biological sciences, this approach has several limitations, especially for data analysis through a homology-based approach (Prakash and Taylor, 2012). Usually, the functional annotations from metagenomic sequences are reached by homology-based approaches to a publicly available reference sequence (Prakash and Taylor, 2012). Although there are numerous pipelines dedicated to functional analysis of metagenomic data, these pipelines can only detect previously characterized genes that are similar to the newly identified genes, and therefore unknown novel bioactive molecules are missed. For this reason, activity-based functional screening of metagenomic libraries is sometimes still the most appropriate way to identify and characterize resistance genes and antimicrobial compounds (Su et al., 2014) rather than searching for genes in public sequence bases. Nevertheless, using this activity-based approach, the genes that have been discovered are evolutionarily distant from known resistance genes in the public databases (Su et al., 2014).

This review will focus on soil samples, which are characterized as a complex and dynamic environmental system, comprising higher microbial diversity of *bacteria*, archaea, fungi, viruses, and protozoa (Young and Crawford, 2004), when compared to other natural environments such as freshwater or extreme habitats (Sleator et al., 2008).

The ecology and activity of soil microbial communities depend on biotic or/and abiotic factors such as soil pH, nutrient availability, water availability, and vegetation cover aboveground (Fierer et al., 2007).

In this context, the present review will attempt a broad literature investigation into diversity and abundance of resistance genes as well as novel antimicrobial compounds, as revealed by the metagenomics approach in the soil microbial community.

## SOIL ANTIBIOTIC RESISTANCE GENES

Although the origin of the genes associated with resistance is considered a mystery, there is a link between antibiotic resistance genes in human pathogens and those found in commensal microorganisms, with several common *bacteria* resistance taxa such as *Staphylococcus aureus*, *Pseudomonas aeruginosa*, and *Klebsiella pneumoniae* coming from the natural environment (Wright, 2010). Generally, *bacterial* resistance to antibiotics can be acquired by horizontal gene transfer (HGT) or by spontaneous mutation in target gene (Hassan et al., 2012). In fact, antibiotic resistance genes could be associated with a transposable element. The mobility of antibiotic resistance genes involves the transference of genetic material to other *bacteria* of the same or different species (Thomas and Nielsen, 2005).

It has previously been reported that antibiotic resistance is everywhere, and consequently efforts are being devoted to understanding the origin of resistance genes, particularly among the vast majority of not-yet-culturable environmental *bacteria*. For instance, the close association between people, animals, and the environment can be responsible for the evolution and spread of antibiotic resistance. For more details, see references (Gautam and Morten, 2014) for an extensive and intense review.

In this context, the environment is constantly exposed to bioactive chemicals produced by a high genetic diversity of plants, protists, *bacteria,* or fungi. For this reason, it is not unexpected that their microbial community possesses specialized mechanisms to respond to and metabolize small molecules, including antibiotics (Knapp et al., 2010), indicating that the environment is a massive reservoir in which to search for novel resistant organisms (Allen et al., 2010). Indeed, the concept of the resistome on a global scale consists of antibiotic resistance genes detected either free living or as commensals in the environment (D’Costa et al., 2006).

Recently, there have been many investigations into resistant microorganisms in natural environments. These environments include deep sea sediments (Song et al., 2005), pristine cave ecosystems (Bhullar et al., 2012), permafrost soil at depths from 2 to 40 m (Mindlin et al., 2008), urban sewage and wastewater (Graham et al., 2011; Novo et al., 2013), and mainly in the human habitat such as the oral cavity (Xie et al., 2010) or gut microbiota (Schjorring and Krogfelt, 2011).

Although a range of environments have been focused, in this review, special attention will be paid to the soil environment because it has been discussed with more detail in the literature. Some recent studies have demonstrated that soil microbial communities serve as a reservoir for understanding the diversity, abundance, and origins of resistance genes (Knapp et al., 2010, 2011; Ma et al., 2014; Su et al., 2014) and that the *bacterial* community is characterized as opportunistic pathogens, including members of the genera *Escherichia*, *Klebsiella*, *Pseudomonas*, and *Streptomyces*, which are known to be multi-drug resistant and ubiquitous in soil samples worldwide (D’Costa et al., 2007).

In this context, the pioneering study carried out by Riesenfeld et al. (2004) was one of the first to use metagenomics to reveal the potential of soil microorganisms in relation to antibiotic resistance genes. This analysis was based on the construction of a metagenomic library from Wisconsin remnant oak savannah soil. Among the gigabase of cloned DNA, nine clones were confirmed to have resistance against aminoglycoside and tetracycline antibiotics (Riesenfeld et al., 2004). Importantly, all DNA sequences from antibiotic resistance genes were significantly different from those previously reported in the database sequences, demonstrating that there was a higher and unexplored *bacterial* genetic diversity in the soil environment.

The same methodology was used in undisturbed Alaskan soil to search for genes that mainly mediate resistance to β-lactam antibiotics (Allen et al., 2009). This analysis was important for future conclusions because the soil that was collected had no previous exposure to antibiotic compounds, never having undergone anthropic pollution. Their results showed that β-lactamases characterized from soil in Alaska were divergent from those isolated in a clinical environment, indicating that they are more related to β-lactamases ancestral homologs (Allen et al., 2009). This finding was attributed to the uncontaminated nature of the sampling site. Although phylogenetic analysis indicated that the β-lactamases sequences from Alaskan soil metagenomic libraries are not related to β-lactamases sequences from the clinical environment, they were still capable of conferring resistance on *Escherichia coli* strains (Allen et al., 2009).

Another example of the characterization of resistance genes in the soil environment was found by Torres-Cortes et al. (2011). Their results showed a high number of clones that conferred resistance to various types of antibiotics. Nevertheless, only one clone screened codified a dihydrofolate reductase denominated *Tm8-3* with approximately 26.8 kDa, which conferred resistance to a synthetic antibiotic such as trimethoprim. In this regard, it was established that the *Tm8-3* enzyme had similar dihydrofolate reductase activity, but with low amino acid identity (41%) against *dhfr* genes, demonstrating an unassigned reductase targeting trimethoprim resistance. The authors emphasize that a functional metagenomics approach enabled them to discover unassigned enzymes with unexpected activities without prior knowledge about their resistance gene sequences (Torres-Cortes et al., 2011).

Although there is no clear evidence if the resistance genes flow from the soil environment to the clinical, or vice-versa, the recent analysis provided by Forsberg et al. (2012) demonstrated that seven resistance gene cassettes from non-pathogenic cultured Proteo *bacteria* from soil conferred tolerance to five antibiotic classes. Additionally these resistance genes had 100% nucleotide identity in relation to those resistance genes detected in pathogens from clinical isolates, suggesting that soil *bacteria* may share antibiotic resistance genes with human pathogens (Forsberg et al., 2012).

It is now becoming accepted that resistance genes are extremely abundant, diverse, and widely distributed in the soil environment, and that they also show clear similarities to those found in common pathogens. All of the studies mentioned above have recovered multidrug resistance cassettes with formerly unknown functions, based on the entries in the GenBank database. Therefore, the vast majority of them were previously identified as antibiotic gene resistance targeting multiple classes of antibiotics, including bacteriocins, β-lactams, aminoglycosides, vancomycin, chloramphenicol, or tetracyclines.

In fact, the main targets of antibiotic molecules are DNA replication, protein synthesis and cell-wall biosynthesis (Walsh, 2000). The knowledge of antibiotic mechanisms of action is extremely valuable for antibiotic resistance prediction. In this context, functional metagenomics investigations have provided a direction for researchers to prospect not only genes that are functionally active against multiple classes of antibiotics in the non-clinical environments, but also to look for genes with unrelated functions that in some circumstances may confer resistance functions (Pehrsson et al., 2013). For example, in the first instance, a metagenomic library derived from South Korean wetland was screened for esterase activity using tributyrin agar plates, which are usually used for detection of lipolytic microorganisms (Jeon et al., 2011). Using functional screening, one clone of the 6912 cosmid clones had previously been screened with esterase activity revealing the presence of an open reading frame (ORF; *estU1*). Although the activity-based functional screening of metagenomic libraries is one quick approach to identify clones with a target of interest, it is important to note that a main limitation of this approach is that it requires expression of the function of interest in the host cell (Schloss and Handelsman, 2003), and for this reason, the frequency of active clones could be quite low.

Despite the low frequency of positive clones, the only positive clone identified in the metagenomic library derived from South Korean wetland was overexpressed in *E. coli,* and the amino acid sequence analysis of EstU1 demonstrated the conserved S-X-X-K motif that is typical of family VIII carboxylesterases.

Furthermore, it had previously been reported that family VIII carboxylesterases and class C β-lactamases are phylogenetically related and both are involved in cleaving the lactamic acid ring, conferring *bacterial* resistance. (Wagner et al., 2002). For this reason, more detailed analysis of purified EstU1 protein showed that this single enzyme has both esterase and β-lactamase activities (Jeon et al., 2011) indicating that EstU1 was the first case of a single enzyme that exhibited notable catalytic features, cleaving the β-lactam ring of antibiotics as well as ester substrates.

In particular, functional metagenomic studies offer alternative opportunities to identify new genes and pathways that produce bioactive molecules. However, there are several technical difficulties in the construction of metagenomic libraries, including bias in the DNA extraction, size of inserts and choice of vectors (Delmont et al., 2011a). In fact, direct or indirect techniques used to extract DNA from soils are not completely efficient, due to the fact that different DNA extraction protocols can yield substantially contrast results with respect to purity, quantity, and high-quality DNA recovery (Delmont et al., 2011b). In relation to the size of the inserts, the metagenomic library can be classified into large-insert (>40 kb) or small-insert (<15 kb) depending on higher molecular weight of DNA. The advantages and disadvantages for each method were discussed in (Rajendhran and Gunasekaran, 2008).

Although the use of *bacterial* host has enabled great progress in biological activities from natural environments, the DNA fragments from other microorganisms such as fungi can be challenging to express in a *bacterial* host (Rondon et al., 1999). This problem is caused by incompatibilities between fungal and host molecular biology, such as the presence of introns in fungal genes and the need for protein glycosylation, which is important for visualizing the target activity. However, in order to overcome these obstacles, extensive efforts have been made to use *Streptomyces lividans* and *P. putida* as alternative hosts (Martinez, 2004).

## NGS AND AMP/ANTIBIOTIC RESISTANCE GENES PROSPECTION

Since the price of sequencing has fallen with the advances in sequencing technologies, researchers have sought to discover alternatives for analyzing such a large amount of available data. Sequencing of the whole metagenome provides information about the functional potential in the environment and intraspecific variations (Schloissnig et al., 2013). Venter suggested sequences assembly as an alternative to identify new genomes in environmental samples (Rusch et al., 2007). Metagenome assembly is a critical step, since researchers are dealing with an unknown number of different genomes, and the possibility of assembling a chimeric sequence is real. It is well known that NGS platforms produce shorter reads than traditional dideoxynucletide sequencing, and short reads are more difficult to assemble, especially for metagenomics (Raes et al., 2007; Kunin et al., 2008). In order to minimize the effect of this sequence mosaic, bioinformaticians have been dedicated to discovering new assembly algorithms and pipelines, which will now be discussed.

MetaVelvet (Namiki et al., 2012) is an extension of the Velvet de novo assembler (Zerbino and Birney, 2008), which uses de Bruijn graphs to connect short reads in a high coverage to construct the contigs. The extension decomposes the graph built by Velvet into individual subgraphs and then assembles each of them separately. Using this approach, the tools can build longer scaffolds and improve the gene prediction. At a species level, MetaVelvet can cover 94.56% the total metagenome size. This is an improvement when compared to the regular Velvet coverage (60.29%) and SOAPdenovo (84.62%). The assembly procedure also leads to better gene prediction: 81842 genes predicted from MetaVelvet scaffolds, 38445 and 65176 predicted genes from Velvet, and SOAPdenovo scaffolds, respectively, (Namiki et al., 2012). Recently, new powerful pipelines to perform the full analysis have been released. Among them we highlight MOCAT (Kultima et al., 2012) and MetAMOS (Treangen et al., 2013). MOCAT is a modular pipeline developed for the processing, assembly, and gene prediction of metagenomics NGS reads. The first step – quality filtering – is essential to avoid assembly errors related to low quality reads. The scaffolds are built using SOAPdenovo (Li et al., 2010), and in the next step the reads are mapped to the assembled sequences. This remapping procedure resolves the chimeric regions and improves gene prediction. MetAMOS has a similar approach, allowing the user to choose from 20 different analysis tools. This tool also provides a functional annotation step that uses BLAST (Altschul et al., 1997) to annotate the predicted ORFs.

Gene prediction is an important step for metagenomics analysis. Identifying ORFs in mixed environment sequences can be a challenge. New prediction tools have been released to help bioinformaticians to identify genes accurately: MetaGUN (Liu et al., 2013) based on Support Vector Machine; MetaGeneMark (Zhu et al., 2010) uses Hidden Markov Models; Glimmer-MG (Kelley et al., 2012) with Interpolated Markov Models. The use of different approaches to reach consisent results increases the accuracy of gene prediction. Gene prediction will provide a set of candidates that can be tested to identify compounds of biotechnological interest. This procedure was used to describe the human gut microbial gene catalog with 3.3 billion different genes (Qin et al., 2010). Among all these predicted genes we can scan for a specific activity.

The characterization of the predicted genes is the most important step toward identifying new antimicrobial peptides and antibiotic resistance genes. To achieve this goal it is essential to use a good reference database for the annotation process. Gene sequences can be retrieved from databases such as NCBI RefSeq (Tatusova et al., 2014) or from secondary databases such as Antibiotic Resistance Genes Database (ARDB; Liu and Pop, 2009). ARDB is a manually curated database that collects information about antibiotic resistance from most of the public databases. The database organizes the information about 23137 genes into 380 different types (e.g., Beta Lactamases, Multi Drug Transporters). Each gene is also related to at least one of 249 antibiotics described in the ARDB and linked to 1737 species. ARDB comprises data about mechanism of action, ontology, orthology groups, and conserved domains. The gene sequence is provided as well, and this information can be used as a reference for sequence similarity searches to infer homology to genes predicted in the metagenomic sample. Besides the characterization of the resistance genes, it is also important to identify nucleotides or amino acid variations and link them to the resistance profile.

Among the metagenomics sequences the identification of genes codifying antimicrobial peptides could also be performed. This identification follows two steps: a sequence similarity search using a reference database, and discovery of novel peptides. A secondary database containing curated peptides with antimicrobial activity can be used for a search using BLAST (Altschul et al., 1997). A profile search can also be performed using HMMR (Eddy, 2011) and the reference database. An example of a database that can be used is APD2 (Wang et al., 2009), which organizes known antimicrobial peptides according to families, sources, and targets. The database contains more than 2400 identified compounds: 1986 have antibacterial, 890 antifungal, and 148 antiviral activities. These data can be used as a reference to assign antimicrobial activity to a predicted gene.

In the soil environment there is much information to be discovered. Due to the lack of reference genomes and curated sequences in databases it will not be possible to classify all the predicted genes using similarity searches. Models generated by support vector machines can be used to classify antimicrobial peptides that are not present in reference databases. AMPPred (Porto et al., 2012) is a software that uses five features to predict novel antimicrobial activity from the translated gene sequences. Using the combination of described tools it is possible to extract the maximum information from one’s data, and characterize antibiotic resistance mechanisms and antimicrobial peptides.

## ANTIMICROBIAL COMPOUNDS FROM SOIL

In addition to the efforts of metagenomics to identify resistance genes that are functionally active against multiple classes of antibiotic, another important attribute of this methodology is the ability to identify several small molecules with antimicrobial activities (MacNeil et al., 2001; Gillespie et al., 2002; Lim et al., 2005). The discovery and screening of novel antibiotics has been an enormous and essential task in recent years. Nowadays, antibiotic resistance poses a global threat to public health (Civljak et al., 2014). On the one hand, bacteria have become more resistant to traditional antibiotic compounds, while on the other, various research groups have selected antimicrobials from different sources including microorganisms, plants, and animals (Roy et al., 2013; de Souza Candido et al., 2014). Among them, the microbial community seems to be the most effective, being the source of many antimicrobial molecules that enable them to live in a very competitive microenvironment. Nevertheless, so far only some microorganisms can be easily detected and cultivated. Metagenomics is a remarkable approach to discover novel entities that could act as a repository of unusual antibiotics in complete environments, such as soil, as focused here.

In most habitats, only a small fraction of all existing prokaryotes are acquiescent to cultivation and chemical study. Moreover, there is a strong body of evidence that uncultivated soil diversity characterizes a massive resource of novel biomolecules with biotechnological potential (Wilson and Piel, 2013). In this context, soil *bacteria* seem to be an important source of bioactive natural products for anti-infective discovery. To select such compounds, several cutting-edge technologies have been applied, including metagenomic library construction, heterologous expression, sequencing of soil microbial communities and single-cell methods (Piel, 2011). Indeed, this workflow starts with DNA isolation, which is typically used for DNA library construction in a suitable host such as *E. coli* (Brady et al., 2009). Moreover, screening efforts rise as insert size decreases, since a high number of clones are needed to cover the target genome. However, the construction of large-insert libraries such as BAC (*bacterial* artificial chromosome) vectors is frequently challenging, due to the amount of DNA obtained and the insufficiency of clone numbers (Piel, 2011). After library construction, the next stage consists of clone identification by harboring genes of interest. The most straightforward screens involve detection of clones exhibiting modified phenotypes, which could be color or appearance based, such as inhibition zones near clones growing on fungal (Chung et al., 2008) or *bacterial* (Rondon et al., 2000) development plates.

An alternative method to phenotypic screens could be based on DNA sequence detection. In spite of higher sequencing costs, one significant advantage over the previous strategy is that natural product pathways can be recognized even in the absence of expression in the library host. This strategy was successfully used for identifying giant multimodular polyketide synthase (PKS) and nonribosomal peptide synthetase (NRPS) clusters (Cane and Walsh, 1999). After phenotype identification a large scale sequencing of enriched bacteria and metagenomes for eDNA gene discovery was applied. Additionally, single-cell analysis has emerged as a powerful strategy to investigate environmental *bacteria* (Wang and Bodovitz, 2010). Isolation of individual cells may be attained by micromanipulation (Frolich and Konig, 1999), flow cytometry (Muller and Nebe-von-Caron, 2010) or microfluidic devices (Weibel et al., 2007). One distinct single cell approach benefit is that genes can be associated with taxonomic information, which is habitually challenging to achieve with metagenomics. This knowledge is of essential importance, since it might lead to the detection of productive taxa and can be valuable for selecting a suitable expression host for cloned genes.

Among the multiple antimicrobial compounds that may be found in nature, a key subject of antibiotic screening from soil samples is polyketides, which are natural products containing multiple β-hydroxyketone or β-hydroxyaldehyde [-H_2_C( = O)CH_2_CH(OH)CH_2_C( = O)-] functional groups. These natural metabolites include the basic chemical structure of multiple compounds with numerous functions such as anticholesteremic and anticancer agents, parasiticides, immunomodulators, and antibiotics (Weissman, 2009).

**FIGURE 2:**
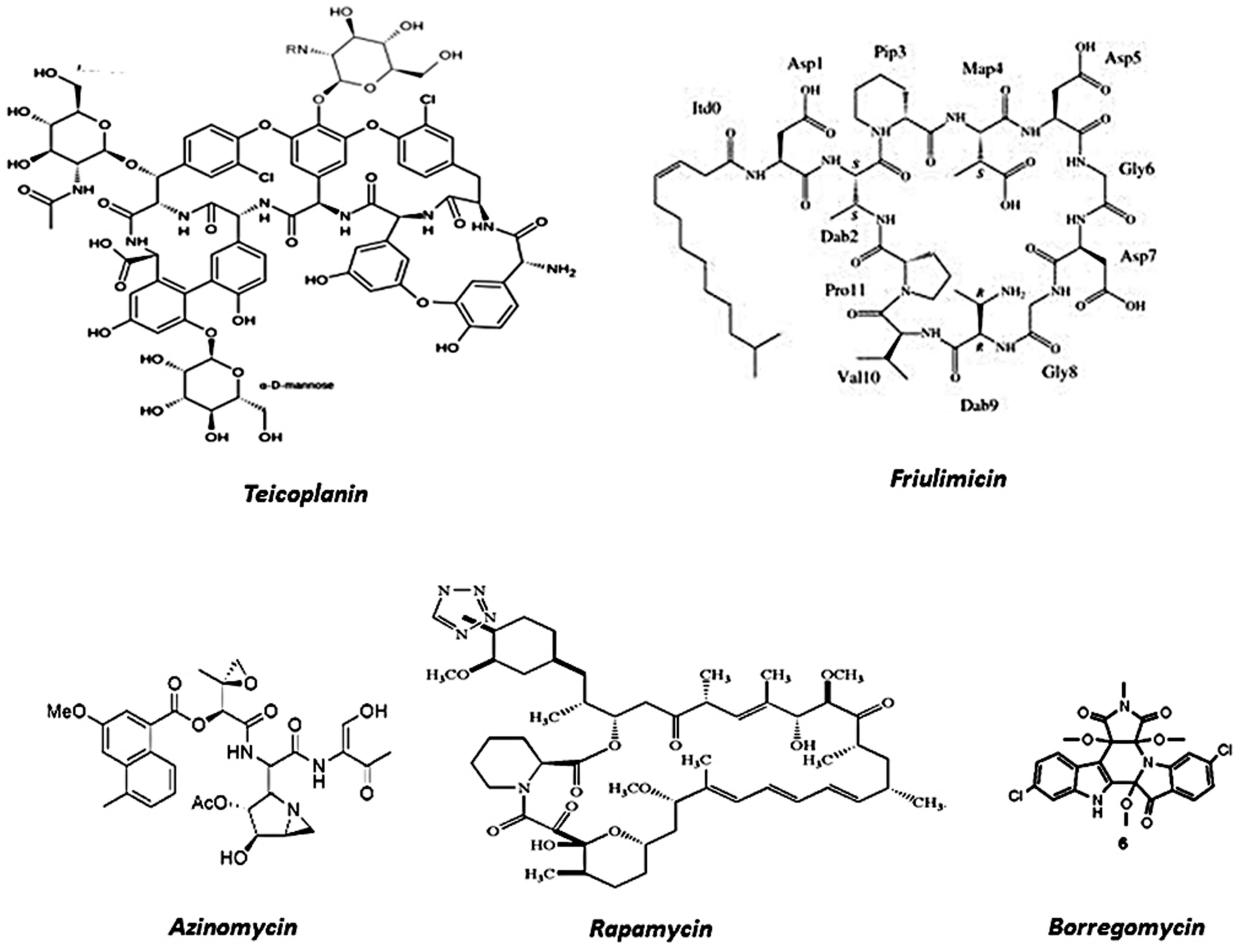
**Prototypes of different antibiotics isolated from soil environments using metagenomic technology**.

Since antimicrobial polyketides were discovered, pharmaceutical companies have invested millions of dollars in the search for potent and selective molecules, which have already included geldanamycin, doxycycline, azithromycin, and erythromycin (**Figure 2**; Robinson, 1991). Polyketides are regularly biosynthesized through the decarboxylative condensation of malonyl-CoA derivative extender units in a comparable fatty acid synthesis process. The polyketide chains synthesized by a PKS are frequently modified into the final version of bioactive natural compounds (Gomes et al., 2013).

The potential of polyketides was immediately associated with metagenomic techniques in order to explore uncultivable soil microorganisms as a remarkable polyketide reservoir (Feng et al., 2011). In order to find novel compounds with biotechnological compounds, (Owen et al., 2013) focused their efforts on a soil sample from the Chihuahuan Desert in southwestern New Mexico. With this aim, conserved regions of PKS and also NRPS biosynthetic machinery were selected to be the target sequences in an experimental framework. By using a combination of PCR amplifications and multiple bioinformatics tools, including AntiSMASH (Antibiotics and Secondary Metabolite Analysis Shell), the authors were able to identify the gene clusters of biosynthetic pathways encoding novel derivatives of therapeutically applicable *bacterial* natural products (Owen et al., 2013). Amongst those with antitumoral action are thiocoraline-like and tallysomycin-like compounds, as well as several antibiotics including two forms of teicoplanin-like, azinomycin-like, two forms of friumilicin-like and a rapamycin-like product. Moreover, in the same report, one glycopeptide yielded from analyses was expressed in order to complete the process of drug production, clearly showing that soil samples could be evaluated by an efficient data-generation pipeline associated with software tool analysis.

Similar analyses were performed in soils sampled from 96 locations throughout the southwestern and northeastern USA, considered the most biologically diverse regions in that country (Charlop-Powers et al., 2014). Once more, PKS were focused and a wide range of antibiotic gene clusters was also found. In this case, not only were the compounds focused but also a chemical biogeographic distribution map of medically valuable groups of natural biocompounds, demonstrating once more the potential of metagenome technology (Charlop-Powers et al., 2014).

Additionally, soil DNA isolated from the Anza-Borrego Desert in California was used to select an indolotryptoline-base for a biosynthetic gene cluster, encoding borregomycins (**Figure 2**; Chang and Brady, 2013). A similar approach was also applied to an antitumor substance BE-54017, also from an indolotryptoline gene cluster (Chang and Brady, 2011). Borregomycins may be yielded from a branched tryptophan dimer biosynthetic metabolic pathway, in which one branch generates borregomycin A, an indolotryptoline antiproliferative agent with the capability to inhibit kinase, and a second branch leading to borregomycins B–D (2–4), which are dihydroxyindolocarbazole agents with anticancer and bactericidal activities. Moreover, BorR clusters were cloned in *Stryptomyces albus* and borregomycins were produced and functionally characterized, clearly showing the complete viability of the metagenome for antibiotic screenings.

Finally and no less importantly, efforts have been made to isolate useful variants from plantaricins. In a study realized by (Pal and Srivastava, 2014), gene-specific primers for plantaricin was used, followed by PCR amplification to select plnE, -F, -J, and -K structural gene amplicons from the soil DNA metagenome. Plantaricin is a cationic, heat-stable peptide belonging to the bacteriocin group (Moll et al., 1999), which is considered a possible alternative to traditional antibiotics against pathogen targets (Cotter et al., 2013). Thus, based on the heterologous production of recombinant plantaricin peptides, the work of (Pal and Srivastava, 2014) demonstrated that plantaricin mature peptides have wide antimicrobial activity spectra, being active against different pathogenic *bacterial* species (Pal and Srivastava, 2014). Plantaricins are short AMPs with the ability to permeate membranes, being extremely dependent on membrane lipid composition. Plantaricins are capable of controlling Gram-positive and -negative bacteria, as well fungi and tumor cells, binding to zwitterionic monolayers and liposomes without significantly penetrating their membranes. Although cholesterol attenuates peptide activity, the association of plantaricin and negatively charged lipid membrane is evident. Furthermore, plantaricins were also able to lead to clear liposome aggregation, suggesting a clear lipid binding mechanism (Zhao et al., 2006). It is important to note that, in accordance with this methodology, it is also possible to achieve a significantly enhanced yield of plantaricin peptides when compared with the purified plantaricin peptides obtained from culturable *Lb. plantarum* strain growth (Pal and Srivastava, 2014), indicating that this methodology can offer an alternative strategy for large-scale production of useful molecules from the natural environment with potential applications in clinical biotechnology research.

## BIOTECHNOLOGICAL PROSPECTS AND CONCLUSIVE REMARKS

The problem of bacterial resistance and the absence of novel antibiotics are being felt on all the continents. Our age has been described as a “post-antibiotic era,” where people die from relatively simple infections that have been curable for decades. Such resistant pathogens will have devastating implications unless significant action is urgently taken. This situation calls for new approaches to discovering novel antibiotics, and metagenome technology seems to be a useful approach to shed some light on bacterial resistance as well to help mine novel and unusual antimicrobial compounds. Nevertheless, metagenomics is a fledgling area of research, and at the moment only a few reports have reached the point of *in vitro* evaluation (Chang and Brady, 2013; Owen et al., 2013). None have been *in vivo* evaluated in animal models so far, but this is a question of time.

Another important question for the pharmaceutical industry concerns production. Since most antibiotics in the pipeline today have been produced by chemical synthesis, it is desirable to produce microorganisms with the ability to secrete such compounds on a large scale and at low cost. However, several pitfalls have been observed, such as folding posttranslational modifications in the case of antimicrobial proteins and peptides, and the dosage amount for antibiotics that at higher concentrations could kill the *bacterial*-host (Parachin et al., 2012).

Indeed, efforts for the development of novel production systems and fermentation process designs are essential for the establishment of a cost-effective methodology for large-scale production of antibiotics associated with metagenome pipelines. Finally, metagenome screening in soil and other environments must also be performed in order to find unusual compounds and not only molecules from known classes. Of course, this is a vast challenge, but a metagenomic approach could allow us to control the most dangerous resistant and infective bacteria. The contribution of the soil metagenome to this field is clear and, along with other under-explored environments, it might be the source of ground-breaking therapeutic agents in the near future.

## Conflict of Interest Statement

The authors declare that the research was conducted in the absence of any commercial or financial relationships that could be construed as a potential conflict of interest.
